# Allosteric Modulators of G Protein-Coupled Receptors

**DOI:** 10.3390/ijms23062934

**Published:** 2022-03-08

**Authors:** Alexander Shpakov

**Affiliations:** Sechenov Institute of Evolutionary Physiology and Biochemistry, Russian Academy of Sciences, 194223 St. Petersburg, Russia; alex_shpakov@list.ru

The G protein-coupled receptors (GPCRs) are the largest group of membrane receptor proteins that are targeted by more than 30% of drugs. Therefore, the search for selective regulators and modulators of the GPCRs is one of the main focuses of molecular biology, biochemistry, and pharmacology. Each type of GPCR has an endogenous signal molecule (amino acid derivatives, peptide and protein hormones, nucleotides, lipids, etc.) that specifically binds to a high-affinity orthosteric site located within the transmembrane domain or in the extracellular regions of the receptor. This leads to conformational rearrangements of the transmembrane domain and its interfaces with intracellular loops, initiating the interaction of the ligand-activated receptor with heterotrimeric G proteins and/or beta-arrestins. As a result, certain intracellular signaling cascades are activated, mediating the appropriate cellular response to the hormonal stimulus.

It is well-known that most GPCRs cannot interact with one transducer protein (G protein or beta-arrestin), but can interact with several such proteins at once. These proteins may be components of a preactivation heterooligomeric complex including GPCR or may be recruited during receptor activation. Therfore, an orthosteric agonist can activate several signaling cascades, causing a wide variety of cellular responses. For example, the binding of luteinizing hormone (LH) to the orthosteric region of the LH receptor not only stimulates G_s_ protein and adenylate cyclase but also activates G_q/11_ and G_i/o_ proteins and beta-arrestins, which regulate other intracellular cascades and mediate receptor internalization. The relatively low selectivity of orthosteric agonists for intracellular effectors significantly limits their application in medicine. Orthosteric neutral antagonists and inverse agonists cause significant and sometimes irreversible inhibition of basal and/or hormone-stimulated GPCR activity, and their clinical use can lead to serious side effects requiring further long-term recovery and rehabilitation.

It would be surprising if nature did not evolve more complex mechanisms for the regulation and modulation of GPCR activity that allow fine tuning of signal transduction generated by orthosteric agonists. The implementation of these tasks is assigned to allosteric regulation, which is carried out both during the interaction of various endogenous substances (metal ions, lipids, amino acids, peptides, proteins, autoantibodies, etc.) with GPCR allosteric sites, and through the formation of homo- and hetero-oligomeric receptor complexes. The allosteric sites can be located inside and outside the transmembrane domain, as well as in the extracellular and cytoplasmic loops of the receptor. 

Allosteric sites can overlap both with each other and with the orthosteric site, which provides opportunities for potentiating or attenuating the effects of orthosteric and allosteric agonists and also determines the specificity of the allosteric ligand-bound receptor for G proteins and beta-arrestins. Thus, targeted receptor-mediated regulation of intracellular effector systems is achieved, taking into account specific physiological conditions. Allosteric GPCR ligands are divided according to their pharmacological properties into positive (PAM), negative (NAM), and silent (SAM) allosteric modulators, as well as allosteric agonists and antagonists, and some of them can combine the characteristics of a modulator and an agonist (the so-called ago-PAM). 

In the Special Issue “Allosteric Modulators of GPCRs” (https://www.mdpi.com/journal/ijms/special_issues/Allosteric_GPCRs accessed on 1 March 2022), Manuel Grundmann and coauthors provide a comprehensive overview of the allosteric regulation of different types of free fatty acid (FFA) receptors that are activated by fatty acids of different length and hydrophobicity [1]. FFA receptors play a key role in the regulation of many physiological processes, and their impaired activity is one of the main causes of metabolic, inflammatory, infectious, endocrinological, cardiovascular, and renal disorders. In this regard, the development of allosteric agonists and ago-PAM for these receptors is of considerable interest for pharmacology. The authors focus on the allosteric regulation of the most studied FFA1 receptor, which is activated by medium- and long-chain fatty acids and is a target for thiazolidinediones used to treat type 2 diabetes mellitus. The review discusses two different allosteric sites, one of which (the A-site) is located at the interface formed by the outer segments of transmembrane helices 3–5 and the proximal segment of the second extracellular loop, while the other (B-site) is located within the transmembrane domain of the FFA1 receptor and partially overlaps with the orthosteric site. The A-site ligands are partial allosteric agonists (TAK-875, MK-8666), while the B-site ligand is an AP8 compound with the allosteric full agonist activity. For TAK-875, an unusual mechanism of binding to the A-site is proposed, in which TAK-875 penetrates into this site, most likely not through the external entrance to the transmembrane channel but through the lipid layer, between the transmembrane helices of the receptor [1]. Although this review focuses on the allosteric regulation of FFA receptors, it also details the general molecular mechanisms of action of allosteric modulators. The authors discuss the opportunities and advantages associated with allosteric regulators, including their high specificity even for closely related GPCRs, biased agonism, and the physiological nature of allosteric GPCR regulation [1]. 

A review by Jan Yakubik and Esam El-Fakahani describes the role of cholesterol as an allosteric modulator of GPCRs, focusing on cholesterol’s ability to directly bind to cholesterol-specific allosteric sites present in various GPCRs [2]. These sites are located both in the extra- and intracellular segments of the transmembrane channel of the receptor and in its eighth pseudo-transmembrane domain (H8), and they contain consensus motifs for cholesterol recognition. Binding of cholesterol to them modulates the efficacy of the orthosteric agonist and alters its structure-activity profile. Cholesterol can function as PAM (the oxytocin, A_2A_-adenosine, 5-HT_1A_-serotonin and μ-opioid receptors) or NAM (β2-adrenergic receptor) or, depending on the dose, influence the selectivity of signaling (the muscarinic acetylcholine receptors). In addition, cholesterol provides fine-tuning for the regulation of various intracellular targets by orthosteric agonists and controls the GPCR oligomerization, influencing the specificity and efficiency of signal transduction [2].

In this Special Issue, Pedro Renault and Jesus Giraldo propose a new computational approach for the identification and study of allosteric sites in the GPCRs based on their assumption that such sites are strongly associated with those regions of the receptor that undergo the most significant conformational rearrangements upon its activation [3]. Using this approach, the allosteric sites have been identified in the β_2_-adrenergic and M_2_-muscarinic receptors (Type A GPCRs) and in the glucagon receptor (Type B GPCRs) and are shown to be in good agreement with data obtained using other methods. Thus, despite some limitations, the proposed approach can become a useful tool for the search and characterization of allosteric sites in GPCRs, especially in cases where other approaches are not effective or suitable [3]. 

In their article, Kazuhiro Mio and coauthors used diffracted X-ray tracking technique to study the movement of the extracellular N-terminal region of the 5-HT_2A_-serotonin receptor in living cells and its dependence on structural and conformational changes in the transmembrane domain of the receptor [4]. For this purpose, gold nanocrystals were attached to the N-terminal region. It is known that the N-terminal region, whose movement and conformational characteristics have not been studied in most GPCRs, is involved in the formation of extracellular allosteric sites and is responsible for modulating the effects of orthosteric agonists. The authors showed that ligand binding significantly reduces the mobility of the N-terminal region, and this depends on the integrity of the DRY motif located at the interface between the third transmembrane region and the second intracellular loop and forms an “ionic lock” between the third and sixth transmembrane regions. It is important that the DRY motif is the main determinant for the interaction of GPCRs with G-proteins. Thus, the relationship between changes in the structure of the transmembrane domain of the ligand-bound 5-HT_2A_-serotonin receptor and the conformational mobility of its N-terminal region involved in allosteric regulation has been shown. 

Anna Glyakina and coauthors focused on the search for the orthosteric and allosteric sites in types 1 and 6 trace amine-associated receptors (TAAR1 and TAAR6) [5]. It should be emphasized that there has previously been no information about TAARs allosteric sites. Using multiple alignments and molecular modeling, it was found that negatively charged residues of aspartic and glutamic acid located in the extracellular loops of TAAR1 and TAAR6 are the main structural determinants of potential allosteric sites in these receptors and can interact with their positively charged allosteric regulators [5]. 

In their article presented in this Special Issue, Andrey Bakhtyukov and coauthors showed that the new compound TP4/2, a low-molecular-weight thienopyrimidine-based agonist of the LH receptor, which binds to an allosteric site located in the transmembrane domain, stimulates testicular steroidogenesis and spermatogenesis in male rats during five-day administration [6]. On the first day, the efficacy of TP4/2 is significantly lower than that of human chorionic gonadotropin (hCG), but by the fifth day, the differences in these effects disappear. This is due to the maintenance of the LH receptor expression in the testes during treatment with TP4/2 but not with hCG. The TP4/2 remains effective in the treatment of aging and diabetic rats. Thus, the prospects for the use of low-molecular-weight allosteric agonists of the LH receptor for long-term stimulation of steroidogenesis and spermatogenesis, both in the norm and in age-related and diabetic pathologies, have been demonstrated [6].
